# In vitro evaluation of antiviral and virucidal activity of a high molecular weight hyaluronic acid

**DOI:** 10.1186/1743-422X-8-141

**Published:** 2011-03-25

**Authors:** Claudio Cermelli, Alessandro Cuoghi, Monica Scuri, Clotilde Bettua, Rachele G Neglia, Andrea Ardizzoni, Elisabetta Blasi, Tommaso Iannitti, Beniamino Palmieri

**Affiliations:** 1 Department of Public Health Sciences, University of Modena and Reggio Emilia, Modena, Italy; 2 Department of Laboratories, Pathological Anatomy and Legal Medicine, University of Modena and Reggio Emilia, Italy; 3 Department of Biological and Biomedical Sciences, Glasgow Caledonian University, Glasgow, UK; 4 Department of General Surgery and Surgical Specialties, University of Modena and Reggio Emilia, Modena, Italy

## Abstract

**Background:**

hyaluronic acid (HA), a non-sulphated glycosaminoglycan, is present in synovial fluid, vitreous humour serum and many connective tissues. Pharmaceutical preparations of HA are used in clinical practice for wound healing, joint pain, kerato-conjunctivitis, asthma, mouth care, oesophageal-reflux, and gastritis. Moreover, it is used as a filler to counteract ageing and facial lipoatrophy. Our study aims at investigating the *in vitro *antiviral activity of a high molecular weight HA.

**Methods:**

the MTT test was used to rule out the potential toxic effects of HA on the different cell lines used in the antiviral assays. The antiviral activity of HA against Coxsackievirus B5, Herpes Simplex Virus-1, Mumps Virus, Adenovirus-5, Influenza Virus A/H1N1, Human Herpesvirus-6, Porcine Parvovirus, Porcine Reproductive and Respiratory Syndrome Virus was assessed by virus yield assays.

**Results:**

the most effective inhibition was observed against Coxsackievirus B5, with 3Log reduction of the virus yield at 4 mg/ml, and a reduction of 3.5Log and 2Log, at 2 mg/ml and 1 mg/ml, respectively: the selectivity index was 16. Mumps virus was highly inhibited too showing a reduction of 1.7Log at 1 mg/ml and 1Log at 4 mg/ml and 2 mg/ml (selectivity index = 12). The selectivity index for Influenza Virus was 12 with the highest inhibition (1Log) observed at 4 mg/ml. Herpes Simplex Virus-1 and Porcine Parvovirus were mildly inhibited, whereas no antiviral activity was observed with respect to Adenovirus-5, Human Herpesvirus-6, Porcine Reproductive and Respiratory Syndrome Virus. No HA virucidal activity was ever observed against any of the viruses tested. Kinetic experiments showed that both Coxsackievirus B5 and Herpes simplex virus-1 replication were consistently inhibited, not influenced by the time of HA addition, during the virus replication cycle.

**Conclusions:**

the spectrum of the antiviral activity exhibited by HA against both RNA and DNA viruses, known to have different structures (with or without envelope) and replication strategies, suggests a non specific mechanism of action, probably involving cell membrane-virus interaction steps. The results of the kinetic experiments support this hypothesis.

## Introduction

Hyaluronic acid (HA) is a non-sulphated glycosaminoglycan which consists of alternately repeating D-glucuronic acid and N-acetylglucosamine units. A huge variety of HAs, with different molecular weights, has been described, probably retaining distinct physicochemical and biological properties. HA is naturally present throughout all mammalian systems, especially synovial fluid, vitreous humour serum and many connective tissues [1]. Moreover, HA is found intercellularly in connective tissues, such as skin, combined with proteins and chondroitin sulphate, where it fulfills important functions involved in tissue structure maintenance, moisture and lubrication [2].

Initially introduced in clinical practice as wound healing promoter, HA is currently used in many medical and cosmetic fields. Some examples of HA applications include eye drops for kerato-conjunctivitis, intra-articular injections for osteoarthritic joint pain, irrigations for bladder and vaginal chronic inflammatory disorders, tracheobronchial aerosolization for asthma, oral solutions for mouth care or for oesophageal-reflux and gastritis. Besides, HA is commonly used for cosmetic interventions, as a filler to counteract ageing and facial lipoatrophy, especially in HIV patients [3].

There is evidence showing the ability of HA to interfere with viral replication *in vitro*. In particular, the replication of Herpes Simplex Virus type 2 [4], Respiratory Syncytial virus [5] and retroviruses [6] is inhibited by HA, while the Adenovirus (ADV) one results enhanced [7]. Such limited and apparently controversial data demand further investigations in order to better understand the HA biological properties.

In this study we investigated the *in vitro *effects of a high molecular weight HA against a wide group of viruses covering a large spectrum of structural features and replication strategies: ADV-5, Coxsackievirus B5 (COXB5), Herpes Simplex Virus type 1 (HSV-1), Human Herpesvirus-6 (HHV-6), Influenza Virus A/H1N1, Mumps Virus (MV), Porcine Parvovirus (PPV), Porcine Reproductive and Respiratory Syndrome Virus (PRRSV). We observed an antiviral activity against COXB5, HSV-1, MV, PPV and Influenza Virus encouraging the use of such compound as a medical tool in specific clinical circumstances.

## Materials and methods

### Hyaluronic Acid

A high molecular HA (1.800 KD) in powder (IBSA, Istituto Biochimico SA, Lugano, CH) was used. It was dissolved in Minimum Essential Medium (EMEM) at 8 mg/ml solution and sterilized by filtration through 0.45 μm filters.

### Cells and Viruses

The following cell lines were used to cultivate the different viruses: two monkey kidney lines, VERO cells for ADV-5, COXB5, HSV-1, and MV and MARC145 cells for PRRSV; the human T-leukaemia lymphoblast line JJHAN for HHV-6; the canine kidney line MDCK for Influenza Virus; the pig cell line PK15 for PPV. VERO, MARC145, PK15 and MDCK cells were cultured in EMEM added with 10% (growth medium) or 5% (maintenance medium) foetal bovine serum (FBS), penicillin (100 U/ml) and streptomycin (100 μg/ml); RPMI 1640 medium supplemented with 10% heat-inactivated FBS, penicillin (100 U/ml) and streptomycin (100 μg/ml) was used for JJHAN cells. All the cell lines were incubated at 37°C with 5% CO2.

The viral strains of HSV-1, ADV-5, COXB5 and MV were clinical isolates, laboratory adapted through serial passages (>50) on VERO cells. The Influenza Virus strain used was the highly neurotropic cell culture adapted WSN33 strain (A/H1N1). For HHV-6, the U1102 strain (variant A) was employed, whereas the two swine viruses tested were reference strains: NADL-2 for PPV and the ATCC strain (Cat. N° VR-2402) for PRRSV. Batches of each virus were prepared, titrated on the suitable cell line and kept frozen at -80°C until they were used for the experiments.

### Cytotoxicity Assay

The MTT test [8] was used to evaluate the effects of the different concentrations of HA on cell viability. Briefly, serial dilutions of HA from 4 mg/ml to 0.5 mg/ml were prepared in maintenance medium and added (250 μl/well) to 24 hr-old cultures of each line. Each dilution was always tested in triplicate and, in each of the 3 experiments carried out, 3 control wells were included. The plates, with the different cell lines, were incubated at different times: 24 hrs for VERO cells, 48 hrs for MDCK and MARC 145 cells, 72 hrs for PK15 cells and 6 days for JJHAN cells. After incubation with HA, the MTT staining was carried out as previously described [9]. Cell viability was calculated as a percentage of the optical density (OD) of the HA-treated cultures in comparison with that one of the untreated controls (100% viability).

### Assay of Cell Protection from Lysis

Twenty-four hour growth VERO and MDCK cells in 96 well plates were exposed for 1 h to HA (4 mg/ml); then a cell lysis solution Triton X-100 was added to each well at a final concentration of 0.1% and 0.5%. After 5' for 0.1% concentration and 15' for 0,5% concentration, the lysis solution was removed and replaced with fresh medium. The cell viability was measured by MTT test and the survival of HA treated cells was compared with that one of HA untreated cultures. In each assay, cells, not exposed to lysis solutions, were used as controls (100% viability). Three experiments were carried out, each one with samples in triplicate.

### Antiviral Assays

The antiviral activity was ascertained by means of virus yield assays. Twenty-four hour growth cell cultures were infected with the different viruses at the following multiplicity of infection values: for COXB5, ADV-5 and MV 0.1Tissue Culture Infectious Dose 50% (TCID50)/cell; for HSV-1, 0.1 Plaque Forming Unit (PFU)/cell; for WSN33 virus, 0.1 PFU/cell; for PPV, 0.1 TCID50/cell; for PRRSV, 0.1 TCID50/cell; for HHV-6, 0.1 TCID50/cell. After 1 hr adsorption at 37°C, the inoculum was removed, the plates washed with PBS and the different dilutions of HA, in maintenance medium, added to the cell cultures (each HA dilution was tested in triplicate). After 24 hr incubation for COXB5, ADV-5, MV and HSV-1, 48 hr for WSN33 and PRRSV, 72 hr for PPV and 6 days for HHV-6 the plates were frozen and thawed three times and the viral yield was titrated by end-point titration for ADV, COXB5, MV, PPV, PRRSV, and HHV-6 and by plaque assay for HSV-1 and WSN33. As far as end-point titration is concerned, 10-fold dilutions of each cell lysate were seeded on the 24 hr growth cells in a 96-well culture plate. After 3 days (6 days for HHV-6), the viral titre of each sample, expressed as TCID50/ml, was read taking into account the final dilution still showing the typical viral cytopathic effect and the results were elaborated using the Reed and Muench formula [10]. Plaque assays were carried out as follows. The samples from the HSV-1 and WSN33 experiments, serially diluted in 10-fold dilutions, were seeded on 24 hr growth VERO or MDCK cells in 24 well plates. After 1 hr adsorption, the viral inoculum was removed, the plates were washed with PBS and the maintenance medium, containing 0.9% Noble Agar, was added. After 72 hr incubation, the plates were stained with Neutral Red and the plaques counted: the resulting titre was expressed as PFU/ml. The selectivity index (SI) was calculated for each virus inhibited by HA as the ratio between the toxic dose 50 and the inhibiting dose 50. For each virus 3 experiments were carried out.

### Effects on the Antiviral Activity of Adding HA at Different Time Points

Time course experiments were carried out with COXB5 and HSV-1 within a single replication cycle. HA (2 mg/ml) was added, at different time points, within 7 hr for COXB5 and 18-20 h for HSV-1 according to each viral replication cycle. In parallel wells, HA was added together with the viral inoculum (t = 0). The virus yield was assessed by end point titration (for COXB5) or plaque assay (for HSV-1). For each virus, 3 experiments were carried out, each in duplicate.

### Virucidal Activity Assays

The different viral inocula were exposed to HA at a final concentration of 4 mg/ml, for 30' at room temperature and then their residual infectivity was titrated on the suitable cells, as described above. A viral inoculum, treated with medium without HA was used as control for each virus. Two experiments for each virus were performed, each in duplicate.

## Results

### Lack of Cytotoxicity Cell protection from lysis

**Figure 1:**
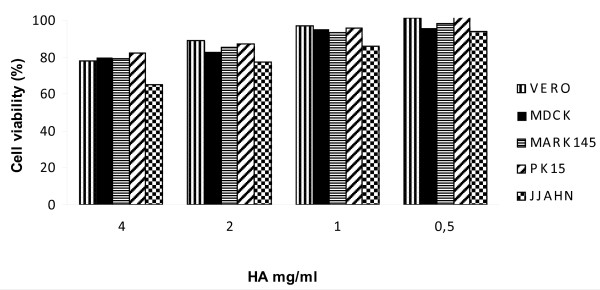
**Twenty-four hour old cell cultures were treated with HA in the maintenance medium and then incubated for different times: 24 hours for VERO cells, 48 hours for MDCK and MARC 145 cells, 72 hours for PK15 cells and 6 days for JJHAN cells**. Then, the MTT assay was performed. Cell viability was calculated as the percentage of optical density of the HA-treated cultures in comparison with that one of untreated controls (100% viability).

Initially, in order to rule out any direct cytotoxic effect of HA, dose-dependent experiments were performed exposing the five cell lines, employed in the experiments, to HA for different times (1 to 6 days, according to the protocols used for virus growth) followed by MTT assay. We found modest cytotoxicity only at the highest HA concentration (4 mg/ml) which caused a OD reduction of about 20% in 4 of the 5 tested cell lines (Figure 1). At the lower concentrations, the OD reduction was about 10% or less. A slightly higher OD reduction (about 30%) was observed on JJHAN cells at 4 mg/ml, probably related to the longer exposition to HA of this cell line (6 days vs 1-3 days for the other cells).

### Cell Protection from Lysis

**Figure 2:**
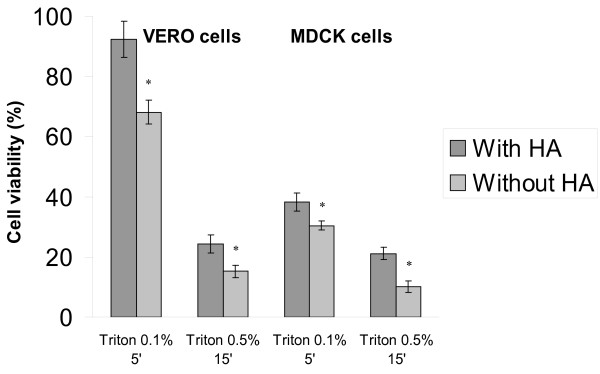
**VERO and MDCK cells, pre-treated or not with HA, were exposed to Triton X-100 at 0.1% and 0.5% concentration for 5' and 15', respectively; then, the cell viability was assessed by MTT assay**. * p < 0.05 with the Student's t test in comparison with HA untreated controls.

With the aim of investigating whether HA may affect cell membrane stabilization, experiments, in which VERO and MDCK cells were pre-treated with HA and then exposed to a lysis solution (Triton X-100) were carried out. We found that both cell lines pre-treated for 1 hr with HA at 4 mg/ml were significantly more resistant to lysis than the untreated controls. In particular, for VERO cells in the HA pre-treated groups, the cell viability was close to 100% with the mild treatment (0.1% for 5') and 24.4% with the stronger one (0.5% for 15'), whereas in parallel groups, not pre-exposed to HA before lysis, the cell viability was reduced to 68.1% and 15.2%, respectively (Figure 2). MDCK cells were more sensitive to lysis treatment, but also in this case the cells pre-treated with HA were more resistant to cell lysis (38,2% and 21.1% the cell viability for the milder and the stronger treatment, respectively) than those not exposed to HA (30.4% and 10.1%).

### Antiviral Activity by HA

**Figure 3:**
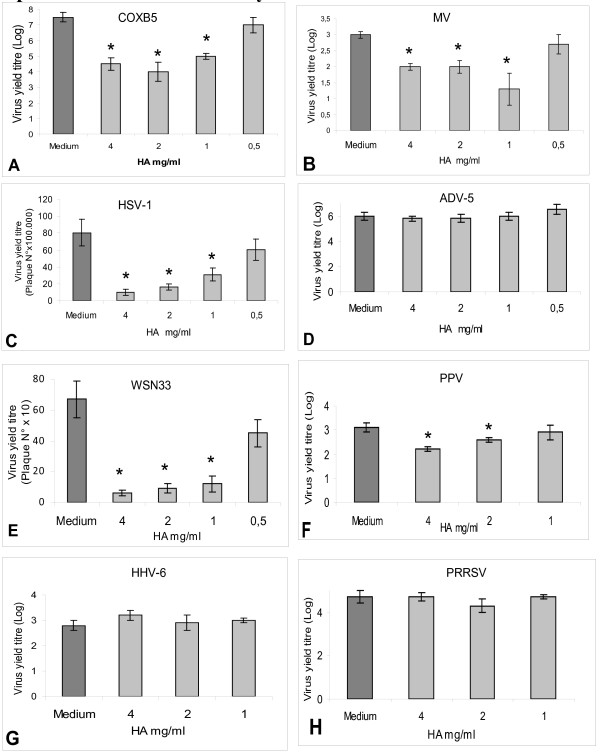
**Different cell lines were infected with COXB5 (panel A), MV (panel B), HSV-1 (panel C), ADV-5 (panel D), WSN33 (panel E), PPV (panel F), HHV-6 (panel G), PRRSV (panel H) and then exposed to HA at different concentrations in the maintenance medium**. The virus yield was titrated by end point titration for COXB5, MV, ADV-5, PPV, HHV-6 and PRRSV (results expressed as Log of the TCID50) and by plaque assay for HSV-1 and WSN33 (results expressed as PFU/ml). * p < 0.05 with the Student's t test in comparison with HA untreated controls.

Virus yield experiments were carried out in the presence of HA to assess its antiviral activity. As shown in Figure 3A, HA exerted the most effective inhibition towards COXB5, with 3Log reduction of the virus yield at 4 mg/ml, and reduction of 3.5Log and 2Log at 2 mg/ml and 1 mg/ml, respectively. This strong inhibition was also confirmed by the SI (Appendix 1) which was 16.1. MV and Influenza Virus were highly inhibited too (SI = 12.1 and 11.9, respectively): 1Log at 4 mg/ml and 2 mg/ml, 1.7Log reduction at 1 mg/ml, for MV (Figure 3B) and about 1Log even at 1 mg/ml for WSN33 (Figure 3E). Two other viruses were inhibited, although to a lesser extent. HSV-1 and PPV showed 1Log reduction only at 4 mg/ml (Figure 3C and 2F) with lower SIs (4.8 and 3.6, respectively). No antiviral activity was observed with ADV-5 (Figure 3D), HHV-6 (Figure 3G) and PRSSV (Figure 3H).

### Effects on the Antiviral Activity of Adding HA at Different Time Points

**Figure 4:**
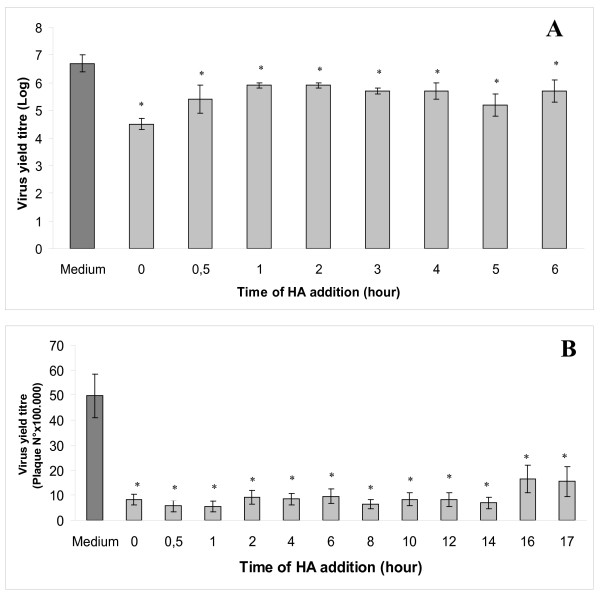
**HA (2 mg/ml) was added at different time points within a single viral replication cycle (7 h for COXB5, 18-20 h for HSV-1)**. In parallel wells, HA was added together with the virus inoculum (T = 0). At the end of the replication cycle, cell cultures were frozen and the virus yield was titrated by end point titration (COXB5, panel A) or plaque assay (HSV-1, panel B). The results are expressed as Log of the TCID50 in panel A, as PFU/ml in panel B. * p < 0.05 with the Student's t test in comparison with HA untreated controls.

In order to investigate the timing of HA inhibition on replication cycle of HSV-1 and COXB5, kinetic experiments were designed. HA was added to the infected cells at different time points, during a single replication cycle. Both COXB5 (Figure 4A) and HSV-1 (Figure 4B) growth were consistently inhibited by HA, regardless of the time of addition.

### Lack of HA Direct Virucidal Activity

With the aim to assess whether HA could directly inactivate virus particles, each viral inoculum was resuspended with HA for 30 minutes before cell infection. The results of these experiments showed that the virus titre was not significantly reduced (<0,5Log) after HA treatment, indicating no virucidal activity by HA against any of the tested viruses (data not shown).

## Discussion

This paper provides a new information on the biological properties of HA, describing its antiviral in vitro activity against 5 viruses, with different structural and biological features.

HA is a non-sulfated glycosaminoglycan widely present in the extracellular matrix of soft tissues and in several biologic fluids [2]. Experimental and clinical data demonstrate the involvement of HA in structure maintenance, moisturizing, tissue lubrication and wound healing. These properties, associated with an excellent safety profile, are exploited in medical practice as well as in aesthetic and cosmetic fields [2,3]. Furthermore, initial evidence also ascribes antimicrobial properties to HA [11-13], adding further appeal to the HA-containing products because of the beneficial effects probably deriving from its antimicrobial properties. Here, we have demonstrated a wide spectrum antiviral activity of a high molecular weight HA. The most effective inhibition is observed against COXB5, MV and Influenza Virus WSN33 (A/H1N1) and it is interesting that the phenomenon is retained even at a concentration (1 mg/ml) lower than that one commonly used for clinical or cosmetic applications. The high SIs displayed by HA for these viruses document a strong antiviral activity not related to cytotoxicity. HSV-1 and PPV are also inhibited by HA, but only at the highest concentration (4 mg/ml): the SIs are consequently lower. No activity is ever observed against ADV-5, HHV-6, PRRSV. The HA failure to inhibit ADV is not unexpected since, according to Chaudhuri [7], HA enhances ADV replication *in vivo *and, though to a lesser extent, also *in vitro*. The different cell system we used may account for the lack of replication enhancement observed in our experiments.

Our findings provide evidence on the effects of HA on a variety of RNA and DNA viruses, with or without the lipidic envelope, characterized by very different replication strategies. Therefore, we can speculate that HA antiviral mechanism(s) probably involves general/non-specific host cell-virus interaction at membrane level, such as virus entry or release, rather than restricted, virus-specific events occurring inside the cell. This speculation is supported by the kinetic results showing that both COXB5 and HSV-1 are growth inhibited irrespective of the time of HA addition during the virus replication cycle. In line with this hypothesis, we may suggest that HA, known to have a heavy ionic charge, may alter the electrostatic interactions between virus and cell receptors and/or other cell membrane components, thus in turn affecting virus entry and/or exit. Literature data substantiate this hypothesis. Dengue virus uses heparan-sulphate (HS) as a cell receptor: different types of HA, interacting with the virus envelope glycoprotein, responsible for virus attachment to HS, are able to inhibit virus attachment and entry [14]. HSV-1 recognizes HS as a receptor too and it has been reported that molecules, affecting the interaction between HSV-1 envelope glycoproteins and HS, are able to reduce the virus growth [15-17]. Several Enteroviruses (including some Coxsackieviruses) are reported to interact with HS for virus entry [18-20]. The results of our kinetic experiments seem to suggest that virus release is also inhibited by HA. Notoriously, this step involves host cell membrane. HSV-1 and COXB5 are released by different mechanisms: HSV-1 by trans-membrane trafficking of vesicles, while COXB5 by cell membrane lysis. Therefore we may assume that the mechanism, by which HA inhibits virus release, has to be non-specific. The results observed in the assay of cell protection from lysis, showing that cells exposed to HA are more resistant to cell lysis, suggest that HA stabilizes cell membranes. This modification could impair any membrane involving process, such as envelope fusion with cell membrane, vesicle fusion and membrane disruption. We have observed that in the antiviral experiments with MV, a syncytiogen virus, not only virus yield was reduced in the presence of HA, but syncytia size also appeared at light microscope observation smaller than those ones of the control cultures. This HA stabilizing activity on membranes indirectly implies that cell exposure to HA inhibits virus entry and/or exit. Finally, the lack of appreciable virucidal activity by HA, against all the viruses under study, rules out the possibility of a direct virus inactivation by HA and it also supports the idea that the steps, involving virus-cell membrane interaction, are preferentially, if not exclusively, affected by HA.

In conclusions, our study provides a wide spectrum demonstration of the antiviral activity by HA, opening new perspectives in prophylaxis and therapy of some viral diseases. The hypothesis of specifically counteracting cell-virus attachment and viral release, by local administration of HA, is very appealing especially for oral, genital and ano-rectal anatomical areas, where compound(s) can be administered as creams, gels or wash solutions. Many HA-based commercial products already available for topic use have HA concentrations much higher or equal to those we found active against different viruses: so it can be hypothesized that effective concentrations can be locally reached. The inhibitory activity observed against HSV-1 is particularly interesting since HA is the basic component of mouthwash solutions and ophthalmic drops, used as artificial tears. According to our findings, HA may be also considered/included as an antiviral agent in the treatment of HSV-1-associated stomatitis and kerato-conjunctivitis in this kind of preparations. Moreover, since Enteroviruses are often responsible for a childhood form of vesicular stomatitis as well as respiratory diseases, the present findings on Coxsackievirus inhibition may open new perspectives for oral administration of HA as natural atoxic medical treatment of newborns and babies. Similarly, the anti-influenza virus activity observed might be exploited in nasal sprays to locally reduce viral replication. Moreover, HA has been demonstrated to have pro-inflammatory activity. Low weight HA fragmentation products and, in the presence of IFNγ, even high molecular weight HA molecules can also activate innate immunity mechanisms through Toll-like Receptor 4 and CD44 [21,22]. This pro-inflamamtory activity may contribute to counteract virus replication and spread *in vivo*.

In conclusion, our findings strongly support the use of this safe glycosaminoglycan in clinical practice as a potential antiviral compound, both for disease prevention and treatment. Further clinical trials on this topic are required to better understand the antiviral activity of this compound.

## Abbreviations

ADV-5: Adenovirus-5; COXB5: Coxsackievirus B5; EMEM: Eagle Minimum Essential Medium; FBS: Foetal Bovine Serum; HHV-6: Human Herpes Virus-6; HSV-1: Herpes Simplex Virus-1; HS: heparan sulphate; MV: Mumps Virus; PFU: Plaque Forming Unit; PPV: Porcine Parvovirus; PRRSV: Porcine Reproductive and Respiratory Syndrome Virus; OD: Optical Density; SI: selectivity index; TCID50: Tissue Culture Infectious Dose 50

## Competing interests

The authors certify that there is no conflict of interest with any financial organization regarding the material discussed in the manuscript.

## Authors' contributions

The authors hereby certify that all work contained in this paper is original work of CC, AC, MS, BC, RN, AA, EB, TI and BP. The authors claim full responsibility for the contents of the article. The authors contributed equally to this work. This article was not supported by grants.

## Appendix 1 - Selectivity indexes of HA

Coxsackievirus B5 - 16.1

Mumps virus - 12.1

Influenza A/H1N1/WSN33 - 11.9

Herpes Simplex Virus-1 - 4.8

Porcine Parvo - 3.6

Selectivity indexes were calculated for those viruses inhibited by HA as the ratio between the toxic dose 50 and the inhibiting dose 50.
